# From Metabolite to Metabolome: Metabolomics Applications in *Plasmodium* Research

**DOI:** 10.3389/fmicb.2020.626183

**Published:** 2021-01-11

**Authors:** Xinyu Yu, Gaoqian Feng, Qingfeng Zhang, Jun Cao

**Affiliations:** ^1^National Health Commission Key Laboratory of Parasitic Disease Control and Prevention, Jiangsu Provincial Key Laboratory on Parasite and Vector Control Technology, Jiangsu Institute of Parasitic Diseases, Wuxi, China; ^2^Medical College of Soochow University, Suzhou, China; ^3^Burnet Institute, Melbourne, VIC, Australia; ^4^Department of Medicine, The University of Melbourne, Melbourne, VIC, Australia; ^5^Key Laboratory of Arrhythmias of the Ministry of Education of China, Research Center for Translational Medicine, East Hospital, Tongji University School of Medicine, Shanghai, China; ^6^Center for Global Health, School of Public Health, Nanjing Medical University, Nanjing, China

**Keywords:** metabolomics, plasmodium, life stage, antimalarial, mode of action

## Abstract

Advances in research over the past few decades have greatly improved metabolomics-based approaches in studying parasite biology and disease etiology. This improves the investigation of varied metabolic requirements during life stages or when following transmission to their hosts, and fulfills the demand for improved diagnostics and precise therapeutics. Therefore, this review highlights the progress of metabolomics in malaria research, including metabolic mapping of *Plasmodium* vertebrate life cycle stages to investigate antimalarials mode of actions and underlying complex host-parasite interactions. Also, we discuss current limitations as well as make several practical suggestions for methodological improvements which could drive metabolomics progress for malaria from a comprehensive perspective.

## Highlights

- The number and quality of metabolomics-based approaches for malarial research have significantly improved due to either the growing interest on parasite infection or substantial improvements in instrumentation, enabling the comprehensive profiling of *Plasmodium* metabolome from an overall perspective.
- Recent metabolomics-related advances, including studying the basic biology of *Plasmodium* in their vertebrate host, investigating the mode of action and resistance mechanisms of antimalarials, and exploring the link between host and parasite metabolic plasticity, has fueled its application in *Plasmodium*.
- As a functional hypotheses-generating strategy, an updated framework for malarial metabolomics research is necessary, including the assessment of obtained achievements and suggestions for further investigation.

## Outstanding questions

• Given that for *in vitro* culture, the malaria parasites mainly reside inside of host erythrocytes, how can the quality of metabolomics data be improved to reduce unnecessary background noise owing to extremely high sensitivity for metabolomics platforms?
• Can *in vitro* metabolomics analysis for *Plasmodium* provide useful information for *in vivo* studies; if not, are human studies necessary for further investigation and what should be taken into consideration when performing clinical trials?
• What new information can we obtain to understand MoAs for antimalarials through untargeted metabolomics study, and are there any limitations when facing new antimalarials?
• Can obtained results be applied from pre-clinical to clinical scenarios? For example, numerous potential infection-related biomarkers have been revealed by different research, how should a proper interpretation or efficient comparisons be made so that more attention can be paid to those compounds with enough practical value?

## Definitions and Methodology: How Does Metabolomics Fit in With System Biology

**FIGURE 1:**
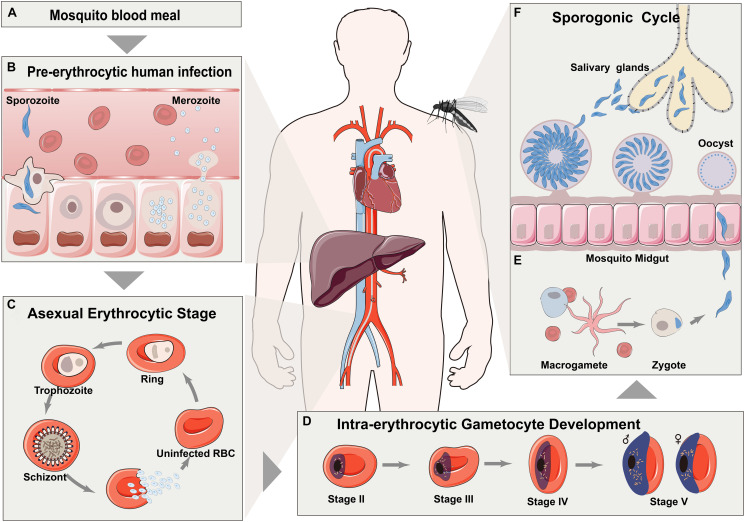
Life cycle of representative plasmodium (*P. falciparum*). **(A)** Malaria is initiated through the bite of *Anopheles* mosquitoes. **(B)** Injected sporozoites are transported to liver through vasculature and then matured in hepatocytes. **(C)** After schizogony by producing hundreds of daughter merozoites, released merozoites then invade into erythrocytes for asexual life cycles. **(D)** A portion of asexually reproducing merozoites undergo reprogramming sexual differentiation to form gametocytes. **(E)** Matured gametocytes are then released into peripheral circulation for ingestion by a mosquito. **(F)** Formed zygotes transform into ookinete in the midgut of mosquito.

**FIGURE 2:**
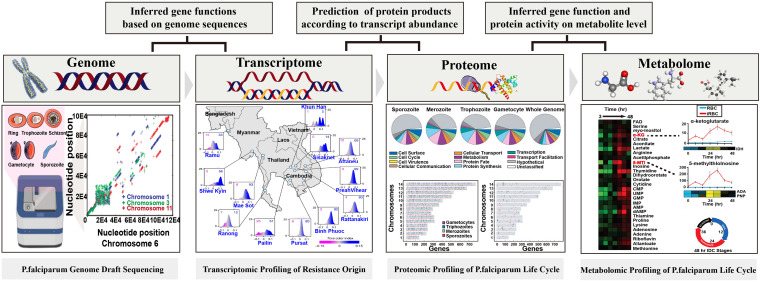
Overview of the relationship between each omics approach and ground-breaking applications in *Plasmodium* spp. Figures of significant achievements are adapted from Gardner et al. (2002), Florens et al. (2002), Olszewski et al. (2009), Zhu et al. (2018) and the copyright of each figure has been achieved.

Malaria has been recognized as a significant global health burden, resulting in approximately 228 million new cases and 405 thousand deaths in 2018 worldwide (Sills et al., 2018; World Health Organization, 2019). Malaria is a vector-borne disease caused by the eukaryotic protozoan parasite of genus *Plasmodium* which has a complicate life cycle, transmitting from *Anopheles* mosquitoes to human hosts (Cowman et al., 2016; Haldar et al., 2018; Figure 1). With the urgent demands for better understanding of the molecular biology of the malaria parasite (Miller et al., 2013), **system biology** has been proven to be a versatile and robust strategy to explore the complex *Plasmodium* biological process (Hasin et al., 2017; Karczewski and Snyder, 2018; Box 1). Advances in these **omics**-based approaches have shed light on parasite biology and further revolutionized the research for parasitic diseases (Figure 2; Florens et al., 2002; Gardner et al., 2002; Zhu et al., 2018).

**Box 1. Systems biology: A comprehensive approach for malarial research.**

Since 2002, the genome draft of laboratory-adapted and field-isolated strains of *P. falciparum* have been sequenced which enables the investigation of epidemiological and transmission dynamics in malaria endemic areas. This excellent work has opened a window for promoting and accelerating drug and vaccine development. However, the large evolutionary distance from model organisms has hindered the genome annotation and the exact functions of specific genes are hypothetical, putative, or even unknown, making it extremely challenging in identifying functional genes in cellular processes. With the growing demands to overcome this challenge, downstream functional omics strategies including transcriptomics, proteomics, and metabolomics have been and continue to be put forward to characterize the gene functions and to obtain better understanding of the molecular biology of malaria parasites (Hall et al., 2005; Salinas et al., 2014; Lindner et al., 2019). Since the first attempt of genome annotation, proteomics research has also been performed to identify stage-specific proteomes or to reveal the critical cellar regulatory mechanisms, suggesting its potential in identification of drug targets or developing transmission block strategies. Meanwhile, the successful construction of various databases, such as VarDB, PlasmoDB, and MalVac, also makes it possible for researchers to share findings, providing broader applications of acquired genomic data to accelerate the development of malaria biology (Chaudhuri et al., 2008; Cristina et al., 2009; Lakshmanan et al., 2011).

It has been well characterized that the epigenetic mechanism or the physiological effect of post-translational modifications can be regulated by **metabolites**. Therefore, changes in the *Plasmodium* development stages can be captured by omics approaches from a genome to **metabolome** level (Skretas and Wood, 2005; Saito and Matsuda, 2010; Sperber et al., 2015). Metabolomics is defined as an approach that aims at simultaneously detecting small molecules (<1,500 Da) to understand the systemic changes in a different state and allows global **metabolic profiling** in various bio-samples. Metabolomics allows the investigation of metabolic phenotypes associated with gene functions and protein expressions by amplifying changes in transcriptomes and proteomes (Saito and Matsuda, 2010; Horgan and Kenny, 2011; Klassen et al., 2017; Giera et al., 2018). Therefore, metabolome can be visually described as metabolic gears intertwined with genomes and proteomes, which acts as an integral component in the overall system without hypothesizing the effect of any single element. Opposed to conventional “down-top” strategies, metabolomics works well in system biology because it is capable of providing one “top-down” view of biochemical profiles in complex organisms. A surge in metabolomics studies were seen over the past decades with the incredible development of high-resolution platforms (Dunn and Ellis, 2005; Markley et al., 2017; Misra et al., 2017; Box 2).

**Box 2. Overview of metabolomics.**

**Analytical Platforms**

Application of various analytical platforms is of extreme importance in which the fast development will greatly aid in our capabilities to understand the biology from an overall perspective. Technological advances will hugely contribute to the detection of thousands of compounds when facing complex biofluids. Historically, nuclear magnetic resonance (NMR) spectroscopy and mass spectrometry (MS) have been the two most widely applied platforms with highly complementary attributes in various metabolomics researches.

Although limited by the sensitivity, NMR still provides a window in the qualitative and quantitative analysis for compounds with high abundance present in almost all biofluids, tissues, or cell extractions. Excellent reproducibility alone with non-destructive property for NMR allows all kinds of sample analysis in a short period, making it an ideal candidate in large-scale metabolomics screening. With the growing demand for capturing low-abundance metabolites to meet the requirement of obtaining metabolic patterns as detailed as possible, the MS platform coupled with chromatography could be another powerful and complementary platform for simultaneous analysis of hundreds of compounds in complex biosamples with sufficient resolution, sensitivity, and reproducibility. Until now, the development trend of analytical platforms still mainly focuses on acquiring more metabolic information in a minimized sample, developing high throughput approaches with improved qualitative and quantitative accuracy.

**Analytical Scope**

• Untargeted metabolomics
  As a hypothesis generating strategy, it mainly focuses on the global assessment of a broad range of both known and unknown compounds to offer either comprehensive or unbiased approaches to investigate unanticipated perturbations. Using this unbiased strategy, changes of metabolic profiles subjected to different conditions can be revealed, leading to the identification of novel metabolites or metabolic pathways. Then subsequent analysis can be performed to structural or functional characterization of these candidates.
• Targeted metabolomics
  As a hypothesis driven strategy, it mainly emphasizes the detection of clearly defined compounds which holds the advantages in obtaining a comprehensive understanding of the kinetic profile in specific pathways.
• Functional metabolomics
  Functional metabolomics, as one kind of emerging concept, spans from the discovery of differential metabolites to investigate the actual function of specific compounds. Based on untargeted and/or targeted approaches, it further characterizes the functional role of selected metabolites as well as the enzymes in related pathways through both *in vitro* and *in vivo* assays including cell biology and clinical platform. It is able to increase the reliability for metabolomics study and contributes novel knowledge about previous findings.

## Metabolic-Based Strategy in Malarial Research: Where Are We Now?

The metabolome is the end-product of intracellular biochemical activities; it is susceptible to changes in the environment, including infection-related stress. Metabolomics studies have been shown to have a critical place in understanding infectious parasite diseases pathology (Kafsack and Llinas, 2010). The complexity of host-parasite interactions resulted from various life cycles, which are often involved in intracellular and/or extracellular stages from insects to vertebrate hosts; considerable efforts have been made to investigate molecular mechanisms to reveal the host immune responses during the invasion and evasion process (Cowman et al., 2016; Gowda and Wu, 2018). However, much less is known about the metabolic interaction and nutrient exchange between parasite and host; therefore, global metabolomics strategy has been put forward for presenting extensive data on *Plasmodium*-infected erythrocytes. Analysis of metabolic profiles for both intracellular and extracellular metabolomes from different life stages is capable of reflecting the perturbations of specific pathways during culture. As clarified, after infection, the parasite will massively convert host metabolome into essential molecules to maintain survival during the **erythrocytic asexual stage**, leading to the excretion of products of erythrocyte lysis and parasite metabolism. Actually, this could have a significant effect on the host response, including events at the cellular or molecular level occurring in both host and parasites (Miller et al., 2013; Cowman et al., 2016). However, complicated interactions between *Plasmodium* spp. and their hosts remains a barrier to complete understanding because of poorly understood downstream consequences, which will be discussed below. In addition to applications on *Plasmodium* biology, continuous work has also been done in the development or screening of effective preventive and therapeutic pharmaceuticals. With the advances in whole-cell screening, thousands of compounds with antimalarial capacity and selectivity have been brought to public attention, providing a promising future in the selection of potent drugs to facilitate the therapeutics pipeline (Gamo et al., 2010). However, one critical bottleneck for this strategy is the lack of mechanistic information about their mode of actions (MoAs). Efforts have been made by genomics, transcriptomics, and proteomics for identifying possible mechanisms from different perspectives, while the emerging metabolomics-based method has been shown to be an attractive strategy due to the metabolic pathway specificity for antimicrobials (Vincent and Barrett, 2015; Dos et al., 2016). Identification of MoA in an untargeted manner can significantly enhance the understanding and detects previously unnoticed pharmacological effects that are ignored by conventional biochemical methods. The unbiased nature, as well as the excellent sensitivity of metabolomics, makes it an ideal approach to map drug-specific metabolic profiles in a time-dependent manner and then to facilitate the understanding of MoA for either commercial-available drugs or novel compounds with antimalarial activities. Therefore, in this review, we mainly aim to outline metabolic achievements in the studies of *Plasmodium* spp. (Table 1), highlighting the promising applications and providing several suggestions for metabolomics research.

**TABLE 1:** Overall view of representative metabolomics studies in *Plasmodium* spp.

Research scope	Analytical platform	Major findings	References
*In vitro* life cycle and host specificity	MS	• Highlight key function of glycerol and glycolysis metabolism in adaption to the variable environments	Mehta et al., 2006; Lian et al., 2009
	MS	• Reveal periodic metabolome and clinically important biochemical pathways during IDC in detail	Olszewski et al., 2009
	MS	• Capture stage-specific metabolic profile and elucidate the metabolomic response to physiologically relevant perturbations	Olszewski et al., 2009
	MS	• Profile detailed arginine depletion and metabolism during the *Plasmodium* invasion	Cobbold et al., 2016b
	MS	• Intergrade unbiased discovery-driven and targeted data-mining strategy to facilitate metabolomic profile	Sana et al., 2013
	MS	• Identify parasite-specific waste products in culture supernatant and confirm disturbed amino acids metabolism	Park et al., 2015; Surowiec et al., 2015
	MS	• Compare metabolic profiles of erythrocyte infected with different strains of P. falciparum	Siddiqui et al., 2017
	MS	• Develop one rapid and easily operated purification method for the enrichment of viable ring stage parasites	Brown et al., 2020
	MS	• Characterize substantial remodeling of mitochondrial metabolism to meet the requirement of gametocyte development	Macrae et al., 2013; Lamour et al., 2014
Mode of action for	MS	• Acquire metabolic fingerprints for commercially available antimalarials and candidates in malaria box	Allman et al., 2016
Antimalarials and	MS	• Provide extensive information regarding the impact of antimalarials on parasite metabolism	Cobbold et al., 2016a
Potential compounds	MS	• Develop medium-throughput and phenotypic screening approach for further mechanistic studies	Creek et al., 2016
	MS	• Report detailed antimalarial susceptibility profiling and reveal stage-specific and metabolic profiles to differentiate MoAs	Murithi et al., 2020
	NMR	• Sheds light on the biochemical changes brought about by genome mutations and its association with drug resistance	Teng et al., 2014
Host-parasite	NMR	• Gain global metabolic pattern for *P. berghei* infected mice and laid foundation for exploring metabolic cross-link in rodents	Li et al., 2008
Interaction and	MS	• Assess the importance of central carbon metabolism and address critical glutamine function in adaptive energy metabolism	Srivastava et al., 2016
Malaria severity	NMR	• Characterize glucose and fatty acids metabolism during the energy exchange with host	Vaughan et al., 2010; Ghosh et al., 2011, 2012, 2016
	MS	• First case-control study to identify candidate molecules associated with pathological process	Abdelrazig et al., 2017
	MS	• Develop metabolomics approach to phenotype patients with varied drug susceptibility and disease severity	Gardinassi et al., 2017; Uppal et al., 2017
	NMR/MS	• Evaluate specific metabolic features associated with CM susceptibility and concomitant metabolic acidosis	Sengupta et al., 2016; Leopold et al., 2019
	MS	• Intergrade metabolomics and transcriptomics approach for discrimination of naïve and semi-immune subjects	Gardinassi et al., 2018

## Laying Groundwork: Profiling From Metabolite to Metabolome

Pioneering efforts were made for the *in vitro* investigation of parasite metabolism early in the 1980s (Zhu, 2004; Macrae et al., 2012; Ng et al., 2014). These studies have reported strikingly distinct metabolic patterns of energy metabolism and has raised the question, how does the parasite adapt to the complex environment by evolving a unique metabolism? Metabolic mapping of *P. falciparum*-infected erythrocytes to track the carbon metabolism has revealed the voraciously consumed level of host glucose, which is almost 100-folds higher than uninfected cells due to the consumption of metabolically-active parasites (Mehta et al., 2006). Surprisingly, glycerol, derived from glucose via glycerol-3-phosphate shuttle, has been proven to serve as an energy source during development, although its origin remains controversial due to the absence of necessary quenching (Lian et al., 2009). Nevertheless, these findings have suggested unique metabolic profiles in parasites which should be further investigated and interpreted with an open mind.

**FIGURE 3:**
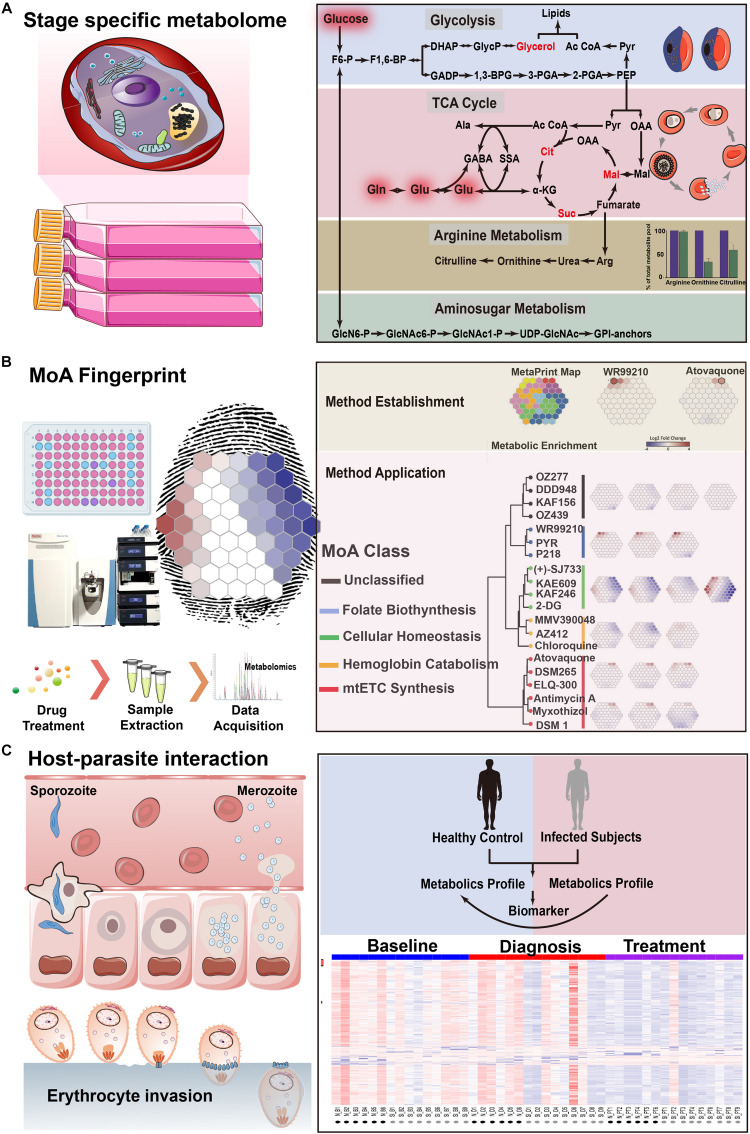
Metabolomics highlights in applications for *Plasmodium* spp. biology**. (A)** An integrated map of an affected metabolic network of *Plasmodium* during different life cycle stages. **(B)** Investigation of antimalarials metabolic fingerprints and mode of action of validated compounds with antimalarial activity. **(C)** Exploration of the link between host response and parasite metabolic plasticity during infection. The copyright of adapted figure has been achieved.

Based on the single metabolite discoveries, successful metabolomics approaches have been performed to map the profile of both parasite-infected host cells and isolated intracellular parasites during life cycles (Figure 3A). Owing to the fact that the blood asexual stage is mainly responsible for clinical symptoms caused by the adhesion of infected erythrocyte, it is of great interest to investigate their metabolomic profile during the intraerythrocytic developmental cycle (IDC). Periodically changed metabolites related to the mature stage parasite (trophozoite and schizont stage) during IDC have been identified, suggesting a close relationship between essential parasite growth and metabolome exchange (Olszewski et al., 2009; Sana et al., 2013; Cobbold et al., 2016b). Most of them are associated with energy metabolism and macromolecular biosynthesis because of excessive metabolic activity for genome replication (Olszewski et al., 2009). Similarly, most prominently alerted glycolysis metabolism has also been revealed by facilitated untargeted profiling and targeted data mining strategies (Sana et al., 2013). In contrast to uninfected erythrocytes with over 90% glucose-to-lactate conversion, disturbed levels of lactic acid resulted from incomplete oxidation suggests both an increased flux of glucose carbon into biomass and the absence of functional tricarboxylic acid (TCA) cycle in energy generation, which correlates well with the minimal oxygen consumption for *P. falciparum in vitro* culture (Brown and Guler, 2020). With the most dramatic change for either accumulated level of non-proteogenic amino acids ornithine and citrulline or sharply depleted arginine in medium (Olszewski et al., 2009), it raises the question for the origin and metabolism of arginine which is not critical for parasite growth. Simultaneous detection of ornithine, citrulline, and arginine by targeted metabolomics has revealed an approximately 1.6-fold up-regulated arginine pool during the asexual stage (especially for trophozoite-stage) resulted from the disruption of nitric oxide metabolism after invasion, proposing one possible mechanism for its metabolism and depletion (Cobbold et al., 2016b). Sufficient information of arginine metabolism, as well as disturbed nitric oxide metabolism, cannot only explain the observed depletion in previous study, but also provide a better understanding for biological events including decreased erythrocyte deformability during parasite invasion. This successful attempt has opened new avenues for capturing a stage-specific metabolic profile in *Plasmodium* and then in turn act as one problem-oriented strategy for malaria research. Additionally, the invasion process has also been profiled by untargeted detection of excretive metabolites in medium supernatants in a time-dependent manner (Park et al., 2015; Surowiec et al., 2015). The positive metabolome-wide association between those shared metabolites with sufficient specificity in ring, trophozoite, and schizont stages has demonstrated the metabolic heterogeneity during development. As noticed, there is an increasing demand for spatially and temporally regulated lipid metabolism resulting from fast production of merozoites with high metabolic activity during growth and differentiation (Gulati et al., 2015). The lipid-associated metabolic pathway has been considered as one attractive drug target, suggesting the importance for comprehensive profiling of lipid metabolism during *Plasmodium* life cycles. Therefore, analysis of either extracellular metabolite levels in medium or intracellular metabolome from different life cycle stages during *in vitro* culture can be applied to infer novel or unanticipated metabolic pathways to functionally characterize the biological process.

As investigation of parasite differentiation is also of practical importance owing to the fact that mature gametocytes are the prerequisite for spread, the metabolomics attempt is then extended to the linear **sexual gametocyte stage** (Macrae et al., 2013; Lamour et al., 2014). Unlike the asexual stage, precise metabolic profiling for gametocytogenesis by tracking the uptake and metabolism of ^13^C-glucose has unexpectedly revealed significantly higher glucose utilization resulted from a substantial remodeling of mitochondrial metabolism (Macrae et al., 2013). This proof-of-principle study has allowed one revision for the energy metabolism of sexual stage parasites which switches into an enhanced TCA-cycle metabolism to meet the requirements of gametocyte development in hypoglycemia. Followed by this explorative attempt, detailed metabolic exchange has been investigated to characterize the gametocyte-specific cell biology by profiling the culture medium (Lamour et al., 2014). Consistent with previous results, most distinct patterns still lie in the glucose utilization as a result of host adaption from the asexual stage to the gametocyte stage. Likewise, the observed sharp depletion of medium-derived lipids associated with gametocyte maturation is also worth further investigation to generate better understanding of gametocyte development, which will facilitate yielding new insights for malaria transmission blocking.

Notably, although capture and detection for metabolome is becoming less of a limiting factor owing to instruments with higher resolution and sensitivity, *Plasmodium* metabolomics studies remain facing the technological challenges to acquire parasites with sufficient homogeneity. Almost all the published results focused on the late-stage parasites which can be efficiently separated from the host cells through density gradient centrifugation or by applying magnetic beads to reduce the background contamination, and these enrichment approaches have greatly fueled recent explosive developments for omics-based approaches in late-stage *Plasmodium* biology (Olszewski et al., 2009; Sana et al., 2013; Cobbold et al., 2016b). Unfortunately, non-specific noise has dramatically hindered our methods to investigate the early-stage parasite, especially in these sensitive down-stream approaches. Although challenging, the enrichment of early-stage parasites with sufficient quantity and purity is of practical value. More recently, one rapid and easily to handle purification method for viable ring stage parasites using a combination of selective lysis property of Streptolysin-O and permeability of Percoll has been proposed. This provides a promising tool for asexual parasite research with good quality (over 80%, 22-fold higher than initial parasitemia) (Brown et al., 2020). Nevertheless, metabolic profiling turns out to be one quality verification experiment for the proposed method. On the other hand, it can serve as one quality control strategy by targeted profiling parasite-specific metabolites that are proportional to the enrichment level to facilitate accurate metabolic investigation of early-stage parasite.

Generally, metabolomics analysis from *in vitro* studies have attempted to build the foundation to capture a stage-specific metabolic profile in *Plasmodium* for a comprehensive understanding of *Plasmodium* biology at a cellular level. Considering that metabolite state varies among different stages of the life-cycle due to parasite evolution (Olszewski et al., 2009; Park et al., 2015), researchers should pay more attention to sample treatment and take both periodic and non-periodic stage-dependent metabolic profiles into account. Dynamic profiling through overall metabolome rather than single target metabolites will aid in understanding how the parasite mediates this exchange progress with host cells. For example, although eukaryotic parasites are capable of *de novo* biosynthesis of some necessary nutrients for growth, it turns out to be more advantageous to utilize host-derived compounds (Müller and Kappes, 2007; Brancucci et al., 2017; Mancio-Silva et al., 2017). One practical challenge during research is the rare homogeneity so that synchronization is critical for isolating specific parasite stage. Nevertheless, it remains a mystery since the nature of intracellular parasitism is nutrient and waste exchange between parasite and host or/and the environment, in which host cells are highly compartmentalized, leaving an inherent difficulty for compartmental analysis of metabolites.

## Finding Networks: Inferring From Antimalarial MoAs to Drug Resistance

With the wide application of effective antimalarials worldwide, malarial control has achieved encouraging success. However, it has been facing extreme challenges with the emergence and spread of drug-resistant strains for most of the commercially available antimalarial drugs, creating a heavy burden for public health and economic development (Menard and Dondorp, 2017). It holds the advantage that understanding of MoA will enhance drug development by monitoring their activities and resistance to facilitate further clinical use, including the optimization of dose and pharmacokinetics, guiding the selection of partner drugs, and strategy development for combating the emergence of resistances (Carolino and Winzeler, 2020). Capturing influenced metabolome may also inform us about the biology of specific strains with varied drug resistance, providing a catalog of candidate targets or pathways to advance critical malarial-control explorations (Figure 3B).

To date, multiple mechanisms of antimalarials have been reported, whereas that of newly available compounds remains yet to be expounded (Olliaro, 2001). The vast majority of them mainly target the essential parasite metabolism to selectively induce lethal outcomes. Thus, metabolomics screening turns out to be an ideal approach to profile these induced perturbations. Until now, applications of metabolomics in the investment of antimalarial MoAs remains largely unexploited. Unlike traditional endpoint assays or phenotype-based methods, which can only provide limited information, metabolomics-based strategies can capture whole-cell metabolic response when facing the treatment of commercially available antimalarials and then be applied for the investigation of related pathways (Allman et al., 2016; Cobbold et al., 2016a; Creek et al., 2016). **Metabolic fingerprint** has been revealed by capturing significantly changed metabolic signatures as well as influenced pathways and, as expected, commercially available drugs (including atovaquone, proguanil, and dihydroartemisinin) displayed distinct metabolic patterns, confirming the feasibility for acquiring unique metabolic perturbation rather than non-specific variance. For example, as a cytochrome bc1 complex inhibitor, disrupted *de novo* pyrimidine synthesis resulted from mitochondrial membrane potential loss has been confirmed by targeting the detection of carbamoyl-L-aspartate and dihydroorotate, the precursor and substrate of affected dihydroorotate dehydrogenase (Cobbold et al., 2016a). In regard to proguanil, a potent prodrug with a poorly understood MoA, significant accumulation of arginine has been observed in which it served as an arginase inhibitor due to structural similarity, suggesting the capability to undercover conventional unknown MoAs with wider perturbations. Although the key mechanism of artemisinin has been characterized, non-specifically released free radicals during the activation process can thus result in multiple effects to the parasite in which more MoAs could be involved in Tilley et al. (2016). Considering the uptake and activation mostly happens in digestive vacuole which results in protein alkylation and the affect hemoglobin catabolism, significantly perturbed hemoglobin-derived metabolism has been observed within 1 h. Unexpectedly, the metabolic profile of dihydroartemisinin also demonstrated the disruption of pyrimidine biosynthesis according to the abundance of isotope-labeled intermediates, even though the specific mechanism differs from atovaquone, indicating dihydroartemisinin could also have an effect on the activity of enzymes such as aspartate carbamoyltransferaseor carbamoyl-phosphate synthase and partly explained the resulted stage susceptibility (Murithi et al., 2020). The proposed method has been extended to screen MoAs for candidates in malaria box, among which 40 of them displayed a drug-like metabolic profile even though they are structurally unrelated. Unlike traditional *in vitro* EC_50_ assay, the proposed method has demonstrated a fast-action manner, suggesting its promising potential in the discovery of fast-acting antimalarial candidates. More recently, another high-throughput metabolomics assay has been put forward for the assessment of stage susceptibility as well as pathway perturbations (Murithi et al., 2020). Likewise, both commercial drugs and antimalarial candidates have been metabolically classified and hierarchically clustered into seven groups to provide a recommended rationale according to their MoAs and drug dynamics. The compounds targeted the same or associated pathways that displayed both similar stage specificity and metabolic profiles; the proposed assay is also capable of differentiating compounds with similar structures. For example, the metabolic profiles of chloroquine and piperaquine, which share the same active structure and displayed similar stage specificity against early ring stages, turn out to be strikingly different, especially for hemoglobin catabolism. From a practical point of view, this research is in favor of the selection of desirable drugs in combination therapies in which combining targets of different life-cycle stages can be applied for optimal therapeutic effect or delaying the emergence of drug resistance.

Likewise, tracking drug-induced perturbation can in turn serve as a powerful tool to profile adapted metabolisms of response phenotypes. Even though genetic or proteomic markers have been regarded as good candidates for monitoring and evaluating resistance, it is still a time and laborintensive task for point-of-care analysis, which is not always suitable for accessing the treatment outcome (Martin et al., 2010; Ariey et al., 2013). However, the exact function of these markers remains unclear from the metabolic level because of the lack of target specificity, suggesting mutations might have a broader impact than expected (Koncarevic et al., 2010; Summers et al., 2012). Thus, global metabolic differences have been characterized between parasites with different susceptibilities for understanding the resistance-related phenotypic diversity (Teng et al., 2014; Siddiqui et al., 2017). Distinct metabolic states for parasites with varied sensitivity have been acquired, especially in amino acid metabolism, phospholipid precursors, and energy metabolism intermediates. The levels of precursors of membrane lipid components or TCA cycle intermediates varied from 5 to 30-fold in resistant strains, suggesting both the membrane lipid and energy metabolism can severe as clues to understanding drug resistance. Another multi-omics integrated attempt has been attempted to enhance the efficiency of metabolomics by associating the decreased level of *PfKelch13* protein with increased levels of glutathione and its precursor, suggesting the perturbation during detoxification enzymes synthesis and regulation (Siddiqui et al., 2017). Hence, metabolomics is able to provide a snapshot of the biochemical status of intercellular activity, telling us how the addressed metabolome changes in response to various factors like drug treatment or adaption to environmental alterations. Moreover, once the responsive metabolome has been identified, the resulted susceptibility or resistance can also be validated by exogenous supplement of targeted molecules.

Currently, the major advantage for metabolomics approaches in the investigation of MoA is the capacity to identify novel drug-related responses at the global level without the need for prior knowledge. Metabolomics has enabled the classification of candidate compounds according to the metabolome-based MoAs, unexpectedly highlighting the effected pathways or enzymes responsible for the antimalarial activity. However, it also brings some limitations; for example, some compounds can induce either a non-specific metabolic response or an indirect effect on the disruption of metabolic pathways. In general, antimalarial candidates in which selected compounds should or at least cover most common MoA of commercial antimalarials to increase the possibility of positive matching. Meanwhile, dynamic time-course or dose-response relationships should also be investigated before screening because it is highly recommended to apply sub-lethal doses which enables the detection of primary effects rather than an undesirable drug-induced death metabolic signature. Based on the above-mentioned criteria, we do suggest that enough replications analyzed in identical conditions are required to ensure the predictability and variation while more quantitative targeted analysis is also required in some circumstances to provide a more detailed pattern of induced metabolome change. At the same time, multi-omics-based approaches also appear to be an attractive and complementary field to profile both primary and secondary responses.

## Extending Applications: Challenging From Pre-Clinical to Clinical

Although *in vitro* parasite culture has been widely explored because of its convenience and maneuverability, it still faces challenges in the absence of a specific immune response and complex nutritional environment. Identifying metabolic perturbations in host **biofluids** is in favor of the understanding of sophisticated host response as well as the development of infection-specific biomarkers. The host-parasite interactions have been extensively reviewed (Kloehn et al., 2016; Ghosh et al., 2018), while the existing challenges are yet to be discussed.

So far, successful attempts have already been made in rodent models (Li et al., 2008; Vaughan et al., 2010; Ghosh et al., 2011, 2012, 2016; Srivastava et al., 2016) and human subjects with symptoms varying from clinically asymptomatic ones to life-threatening ones (metabolic acidosis or cerebral malaria) (Sengupta et al., 2016; Abdelrazig et al., 2017; Gardinassi et al., 2017; Uppal et al., 2017; Gardinassi et al., 2018; Leopold et al., 2019; Figure 3C). Remarkable glycolytic up-regulation and energy demand have been observed due to the parasite metabolism shift from glycolysis (asexual stages) to the enhanced TCA cycle (both gametocyte stage and ookinetes), suggesting an adaptive manner of energy metabolism during the host switch from rodent to mosquito (Srivastava et al., 2016). While in human hosts, metabolomics approaches also showed potential in the differentiation of uninfected controls from infected cases with varying severity by using either relative level changes of specific metabolites or unique metabolomes in infected ones. Likewise, lipids metabolism and amino acids metabolic perturbation (especially for aspartate and asparagine) has been shown to have satisfactory sensitivity in patients. Also, distinct metabolic and transcriptomics patterns for semi-immune subjects has indicated a systemic immune response after secondary infections which further resulted in improved symptoms (minor or no symptoms) (Gardinassi et al., 2018). In addition to this situation, closely related lipid composition and glycoprotein for CM-specific susceptibility also suggests a candidate mode for early diagnosis. Particularly, the temporal metabolic profile of specific organic acids (including hydroxyphenyllactic acid, phenylacetic acid, and dimethylglycine) in patients with metabolic acidosis also proposed one possible mechanism to combine the severity of malaria with the dysfunction of the gut microbiota.

Although remarkable metabolomics-based efforts for the investigation of parasite infections have been able to differentiate infected subjects from healthy controls, these approaches are still inadequate for clinical diagnostic application because sometimes these biomarkers are general indicators of infection rather than disease-specific predictors. There remain some open questions that must be answered in the future. For example, do the host responses exactly reflect the severity? Do the parasites have a unique metabolome in the host with varied symptoms or severities? Does the infected host metabolome actually result from the host-parasite interaction? Therefore, one major challenge for investigating host-parasite interactions mainly relies on the biomarker validation in which the valuable biological meaning is required for detailed mechanisms. In most cases, to ensure so-called “novelty,” there is an absence of functional information for acquired compounds altogether while the biggest hurdle is how to correlate the candidate metabolites with the phenotypes and further move beyond biomarker identification to mechanism investigation. Theoretically, follow-up validation studies should be performed in other cohorts to ensure objectivity. However, this will result in another problem: how to overcome the individual differences that arise from both genetic and environmental factors to point out the biological meaning of the selected metabolome. Therefore, developing proper normalization strategies as well as a useful database is of great help to obtain a metabolic profile detailed to metabolite concentrations ranging from various influential factors.

On the other hand, metabolome serves as down-stream products like substrates or intermediates for various biochemical reactions, suggesting particular sensitivity to their corresponding enzyme activity and abundance. Although quantitative protein analysis, like enzyme-linked immunosorbent assay or immunoblotting, is of lower throughput, they are more straightforward and readily available to profile metabolite-protein relationships which is in favor of providing useful information associated with metabolomics findings. Additionally, high-throughput omics approaches can also be integrated to transform single metabolomics data into an enhanced understanding of the complex biological process. One guilt-by-association strategy has been put forward in which either known or unknown co-expressed genes in one specific metabolic pathway have been investigated with an associated metabolic profile to predict and validate the pathophysiology (Gerth et al., 2016). This attempt sets both a comprehensive and straightforward overview and framework to explore the infected-related metabolic profiling with corresponding immune function, not only in malaria but also in other parasite diseases.

Generally, acquired potential biomarkers from human subjects or animal models can serve as a starting point for mechanistic research to determine exact biological functions for more reliable data. In the near future, more blind studies with larger cohort sizes are required to validate the efficiency of discovered potential biomarkers, yielding reliable diagnostic markers, predictors of treatment outcome, and related clinical polymorphisms.

## Conclusion Remarks

The advances in metabolomics during the past few decades have opened the door for a deeper biological understanding of the critical metabolic profile in *Plasmodium* spp. With some pioneering studies, solid groundwork has been laid to establish links in metabolic networks from specific metabolites to global metabolome. Promising applications have been achieved as reviewed, including investigating varied metabolic requirements during life stages or through following transmission to their hosts; from these efforts, MoAs or targets of various compounds have been identified and the predictive markers of infection defined. Generally, both the obtained stage-specific metabolic profile and the candidate biomarkers from *in vitro* or *in vivo* studies have strengthened the comprehensive understanding at cellular level, potentially severing as a starting point for future investigations to explore the exact biological functions of acquired metabolome. Meanwhile, globally captured drug-related response has turned out to be helpful in the understanding of novel or unexpected MoAs, further facilitating their clinical use, including the optimization of dose and pharmacokinetics, guiding the selection of partner drugs for combating the emergence of resistances.

However, several questions still remain (as shown in Outstanding Questions). For example, neither rare homogeneity during *in vitro* culture nor the difficulty in interpretation and validation for potential candidates from human subjects has greatly hindered the clinical applications of metabolomics-based research. In our opinion, even though the above-mentioned limitations mainly result from intrinsic characteristics of metabolomics, they can be technologically improved through methodological advances and sophisticated study design. Specifically, a shorter synchronization window or efficient enrichment of a particular stage should be performed to obtain parasites with good quality for accurate metabolome acquisition. Meanwhile, sustained effort is highly recommended for the construction of parasite-specific databases or sharing clinical metabolomic samples in both longitudinal and independent cohorts. In addition to target validating, meta-analysis has shown to be another powerful strategy in which multiple studies for one disease or symptom are compared to exclude those unrelated features for the shared phenotype. Considering the unbiased property of metabolomics, in the investigation of MoAs for antimalarials, we do suggest the selected antimalarial candidates to cover the most common MoAs with available compounds to increase positive hits rather than be classified into an unknown group. Further, dynamic time-course or dose-response experiments should be performed before screening to obtain the primary effects.

One current trend toward standardization of varied metabolomics workflows is a practical way to make the technology and the data more accessible. We believe the future of metabolomics in *Plasmodium* research is to be integrated with traditional system biology strategies routinely used in parasitological studies. More system biology approaches will be generate growing interest from genomics, transcriptomics, proteomics, and metabolomics. These combinatorial strategies will allow for better understanding of the biological events from a more global perspective, yielding a detailed view of its intricate networks. Such approaches can undoubtedly inform many aspects of malaria research including, but not limited to, building basic parasite biological models, development of therapeutics, identification of diagnostic biomarkers, and establishment of human pathophysiology models.

## Author Contributions

QZ and JC were responsible for providing the idea and framework of the whole work. XY wrote the first draft of the manuscript. GF did critical revision of the article. All authors contributed to the article and approved the submitted version.

## Conflict of Interest

The authors declare that the research was conducted in the absence of any commercial or financial relationships that could be construed as a potential conflict of interest.

## Glossary

**Asexual blood stage:** one life cycle refers to the *Plasmodium* infection and development in host erythrocytes. Parasite morphologically transition from ring to trophozoite and further to schizont which is capable to release infectious merozoites for invasion.

**Biofluids:** any organism derived bio-organic fluids including blood, urine, saliva, cerebrospinal fluid, etc.

**Erythrocyte:** a set of vertebrate red blood cell.

**Metabolic fingerprint:** high-throughput classification of candidates according to global patterns of captured metabolite signals without exact identification.

**Metabolic profiling:** quantification analysis of whole metabolome (known and unknown) as precise possible to obtain the overall metabolic spectrum with respect to their chemical nature or metabolic pathways in biosamples.

**Metabolites:** small molecules that are biosynthesized, processed, and degraded resulted from cellular metabolic reactions.

**Metabolome:** chemical entities including both primary and secondary metabolites, metabolic intermediates, and signal compounds in biosamples.

**Omics:** an extensive term for strategies aim at exhaustively analyzing whole class of biomolecules including genome, transcriptome, proteome and metabolome.

**Sexual gametocyte stage:** life stage during which a portion of asexually reproducing merozoites undergo reprogrammed sexual differentiation to form gametocytes.

**Systems biology:** comprehensive analysis from overall perspective without only focusing on specific part.

## Abbreviations

F6-P: fructose-6-phosphate
F1,6-BP: fructose-1,6-bisphosphate
DHAP: dihydroxy-acetonephosphate
GlycP: glycerol-3-phosphate
Ac-CoA: acetyl-CoA
Pyr: pyruvate
GADP: glyceraldehyde-3-phosphate
1,3BPG: 1,3-bisphosphoglycerate
3-PGA: 3-phosphoglycerate
2-PGA: 2-phosphoglycerate
PEP: phosphoenolpyruvate
Ala: Alanine
GABA: γ-aminobutyric acid
SSA: succinic semi-aldehyde
Gln: glutamine
Glu: glutamate
αα-KG: α-ketoglutarate
Cit: citrate
OAA: oxaloacetate
Mal: malate
Arg: argine
GlcN6-P: glucosamine-6-phosphate
GlcNAc-6P: N-acetyl -glucosamine-6-phosphate.
